# Significance of aquaporins’ expression in the prognosis of gastric cancer

**DOI:** 10.1042/BSR20171687

**Published:** 2018-06-12

**Authors:** Saroj Thapa, Mandika Chetry, Kaiyu Huang, Yangpei Peng, Jinsheng Wang, Jiaoni Wang, Yingying Zhou, Yigen Shen, Yangjing Xue, Kangting Ji

**Affiliations:** 1 Department of Cardiology, The Second Affiliated Hospital and Yuying Children's Hospital of Wenzhou Medical University, Wenzhou 325000, China; 2 Department of Obstetrics and Gynecology, The Second Affiliated Hospital and Yuying Children’s Hospital of Wenzhou Medical University, Wenzhou 325000, China

**Keywords:** Aquaporins, AQPs, gastric cancer, KM plotter, prognosis

## Abstract

Gastric carcinoma is one of the most lethal malignancy at present with leading cause of cancer-related deaths worldwide. Aquaporins (AQPs) are a family of small, integral membrane proteins, which have been evidenced to play a crucial role in cell migration and proliferation of different cancer cells including gastric cancers. However, the aberrant expression of specific AQPs and its correlation to detect predictive and prognostic significance in gastric cancer remains elusive. In the present study, we comprehensively explored immunohistochemistry based map of protein expression profiles in normal tissues, cancer and cell lines from publicly available Human Protein Atlas (HPA) database. Moreover, to improve our understanding of general gastric biology and guide to find novel predictive prognostic gastric cancer biomarker, we also retrieved ‘The Kaplan–Meier plotter’ (KM plotter) online database with specific *AQPs* mRNA to overall survival (OS) in different clinicopathological features. We revealed that ubiquitous expression of AQPs protein can be effective tools to generate gastric cancer biomarker. Furthermore, high level *AQP3, AQP9*, and *AQP11* mRNA expression were correlated with better OS in all gastric patients, whereas *AQP0, AQP1, AQP4, AQP5, AQP6, AQP8*, and *AQP10* mRNA expression were associated with poor OS. With regard to the clinicopathological features including Laurens classification, clinical stage, human epidermal growth factor receptor 2 (HER2) status, and different treatment strategy, we could illustrate significant role of individual *AQP* mRNA expression in the prognosis of gastric cancer patients. Thus, our results indicated that AQP’s protein and mRNA expression in gastric cancer patients provide effective role to predict prognosis and act as an essential agent to therapeutic strategy.

## Introduction

Numerous studies have presented gastric cancer as one of the major leading causes of mortality from malignancies with most lethal impact on global health. Over the past decades, the 5-year overall survival (OS) rate has been estimated to be more than 90% at early stage gastric cancer, which has been declined to less than 25% with rapid invasion and disease metastasis [1–3]. The advances in adjuvant therapy, surgical techniques, molecular targetted therapy, radio-chemotherapy, and early diagnosis of the disease might have improved the prognostic outcomes of gastric cancer patients [4]. However, the prognostic incidence with advanced disease yet remains worse and unsatisfactory. In order to determine the better outcomes and to establish novel therapeutic targets for refractory gastric cancer, it is urgently needed to identify reliable predictors of bad prognosis or recurrence in patients with advanced gastric cancer. Moreover, gastric cancer prediction for prognosis and early detection also has a lack of proper non-invasive technique [5]. Recent study has reported various oncogenes, tumor suppressor genes, protein and miRNA as the potential gastric cancer biomarkers that are closely related with carcinogenesis, progression, and aggressiveness [6].

Aquaporins (AQPs) consisted of 13 different (AQP0–AQP12) subtypes, which are known as integral membrane proteins, amongst which AQP0, AQP1, AQP2, AQP4, AQP5, AQP6, and AQP8 are primarily water selective, whereas AQP3, AQP7, AQP9, AQP10, and AQP12 (called aquaglyceroporins) are responsible for transporting water, glycerol, and other small solutes like urea [7]. So far, the current researches have shown that AQPs have essential role in cell proliferation correlated with cell functions, for example invasion, migration, angiogenesis, and wound healing through facilitating changes in cell shape under elevated osmotic stress [8]. AQPs are strongly expressed by numerous tumor cells mostly those from malignant tumors with higher metastatic potential and enhanced local infiltration [9–11]. Accumulating evidences have demonstrated that overexpression of AQPs are highly correlated with different gastrointestinal malignancies such as: colorectal cancer [12], esophageal squamous cell cancer [13], and gastric cancer [14]. In particular, Shen et al. [15] stated that differential expression of AQPs are associated with the differentiation, lymphovascular invasion, and nodular metastasis of human gastric carcinoma leading to gastric tumorigenesis. Thus, investigating AQPs activity can reveal a potential universal marker for malignant transformation of gastric cancer. However, prognostic events of specific AQPs protein and mRNA expression in gastric cancer remain elusive. In the present study, we retrieved data on 11 AQPs subtypes from online database to access the effect of the genes on gastric cancer prognosis in different clinicopathology involving histological subtypes according to Laurens classification, clinical stage, gender, human epidermal growth factor receptor 2 (HER2) status, and treatment strategy, generated from Kaplan–Meier plotter (KM plotter) online database (http://kmplot.com/analysis/). Furthermore, to increase the utility of the current proteomic resource, we have systematically integrated our data with a multitude of publicly available Human Protein Atlas (HPA) database. The HPA (http://www.proteinatlas.org/) contain samples from 48 different normal human tissues, 20 different cancer types, 47 different human cell lines, and 12 hematopoietic cell types from patients [16,17]. Annotation of cell lines in HPA is achieved through automated image analysis based on immunohistochemical (IHC) staining, and similarly scoring system has been applied to define the level of expression. The database therefore actively support the potential biomarker with the aim to identify protein expression pattern indicating if specific protein could be used as a biomarker.

## Materials and methods

### The HPA

The HPA (www.proteinatlas.org) contains IHC-based expression data for 20 highly common forms of cancer with 12 individual tumors representing each tumor type [18]. Moreover, the database allows for efforts to identify tumor-type specific expression patterns and also to identify proteins that are differentially expressed in various tumors of a given type. We searched the HPA database for cancer cell-secreted/released protein of interest using an arbitrary selection criteria, for example protein expressed in greater than 50% of the tumor tissue sections were examined. In the IHC image, consecutive sections of human normal and gastric cancer were stained using two different antibodies: HPA and CAB. The standard validations were allowed for direct comparison of different protein expression patterns within the tissue and subcellular compartments. Ultimately, we generated significantly expressed protein results of individual AQP genes in normal tissues and gastric cancer tissues via database and evaluated.

### The Kaplan–Meier survival analysis

The prognostic events of the individual mRNA expression of AQPs in gastric carcinoma was speculated using KM plotter online database (http://kmplot.com/analysis/) [19]. Additionally, we evaluated the correlation between OS of gastric cancer patients and specific AQPs based on histological subtypes, clinical stage, gender, HER2, and different treatment strategy and gene mutation. As we know, OS measurement is the essential technique to define clinical therapeutic success, we therefore pooled the relevant outcomes from the database and assessed all the significantly available *AQPs* mRNA expression and its relation. Currently, potential records of 54675 genes in the effect of survival in ovarian cancer [20], breast cancer [19,21], lung cancer [22], and gastric cancer [23] are available in the database. OS information of 1065 gastric cancer patients in the database were recognized from Cancer Biomedical Informatics Grid (caBIG, http://cabig.cancer.gov/, microarray samples published in the caArray project), the Gene Expression Omnibus (GEO, http://www.ncbi.nlm.nih.gov/geo/), and The Cancer Genome Atlas (TCGA, http://cancergenome.nih.gov) cancer datasets [22].

Collectively, in the present study, we have retrieved 11 members of AQPs (AQP0, AQP1, AQP2, AQP3, AQP4, AQP5, AQP6/2L, AQP8, AQP9, AQP10, and AQP11) entering through the database (http://kmplot.com/analysis/index.php?p=service&cancer=gastric) to analyze Kaplan–Meier survival curves. We could not retrieve data on perforation, differentiation, and TNM stage for the study due to low patient population and unavailable data, which has high probability of confounding results. The expression cut-off points of each AQP genes were determined according to their median mRNA levels from the selected gastric cancer samples. Finally, AQPs expression were categorized into ‘low’ and ‘high’ depending on the comparisons between expression values with established cutoffs. Two levels of an explanatory variable cohorts were compared with Kaplan–Meier survival plot, and then hazard ratio (HR) and 95% confidence interval (CI), as well as log rank ‘*P*’ were measured from the database and displayed. *P*-value of <0.05 was deemed to be statistically significant.

## Results

### HPA

**Figure 1:**
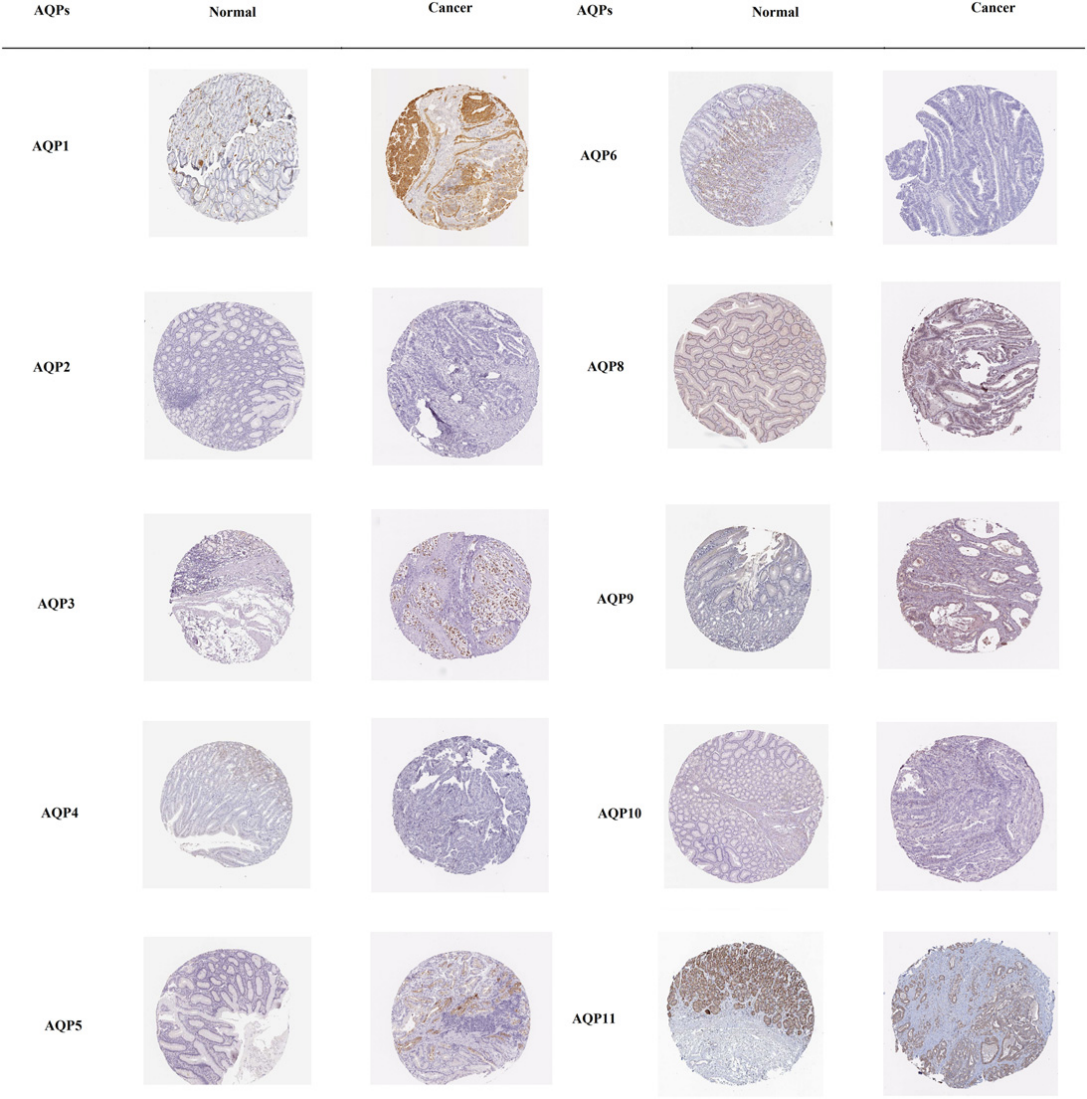
Immunohistochemistry images as obtained from HPA Selected images of proteins detected in HPA database that showed trends toward differential expression in normal and gastric cancer tissues.

Eleven individual AQP family genes protein expression in normal gastric tissues and gastric cancer tissues were selected from the HPA database. We systematically screened the available immunohistochemistry images of all available proteins displayed in the database. Several proteins with intriguing patterns were demonstrated in differential expression in normal gastric compared with gastric cancer tissue although image quality differences were also documented (Figure 1). In the present analysis, we revealed that AQP2, AQP6, AQP9, and AQP10 proteins were not expressed both in normal and gastric cancer tissues. However, AQP1 and AQP8 showed a trend toward decreased expression in normal gastric tissues, whereas its high and medium expression was documented respectively in cytoplasmic and membranous region of gastric tumor tissues. Alternatively, we found an elevated expression AQP4 protein trend toward normal gastric tissues, while no expression was detected in gastric cancer tissues. Intriguingly, we detected strong expression of AQP3 protein both in normal and gastric cancer tissues. Similarly, AQP5 protein had low expression in normal tissues, whereas AQP11 protein had higher expression in normal gastric tissues, but both proteins have medium expression in cytoplasm and membranous region of gastric cancer tissues. Eventually, we did not find any investigations related to AQP0 in the database.

### Different prognostic correlation of AQP subtypes in all gastric cancer patients

**Figure 2:**
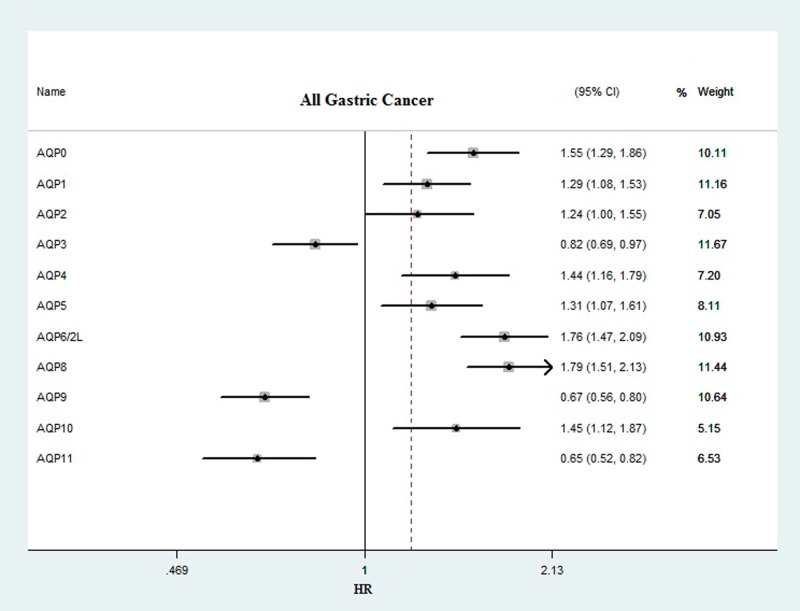
Prognostic values of the *AQPs’* mRNA expression in all gastric cancer patients by using www.kmplot.com

**Figure 3:**
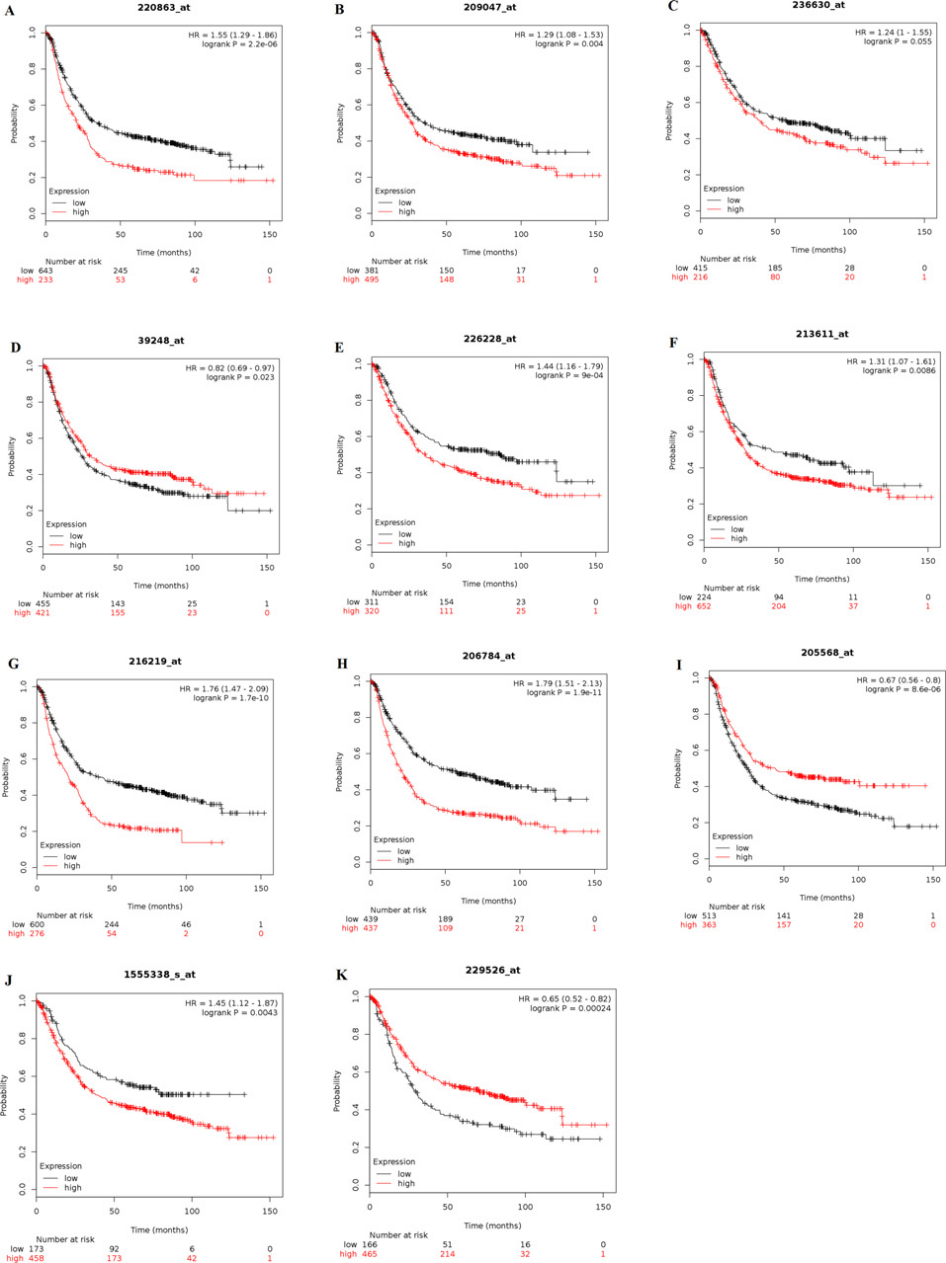
Prognostic significance of individual *AQP* mRNA expression in gastric cancer patients Survival curves of (**A**)AQP0, (**B**)AQP1, (**C**)AQP2, (**D**)AQP3, (**E**)AQP4, (**F**)AQP5, (**G**)AQP6/2L, (**H**)AQP8, (**I**)AQP9, (**J**)AQP10 and (**K**)AQP11 are plotted for all patients (n=876) (www.kmplot.com).

We comprehensively explored prognostic events of the 11 AQP subtypes’ mRNA expression in gastric cancer patients through Kaplan–Meier survival information on www.kmplot.com. Out of 11 members, we accessed 10 members significantly correlated with the prognosis for all gastric cancer patients (*n*=876) (Figure 2). We documented that high level of *AQP0* (The Affymetrix IDs is valid: 220863_at), *AQP1* (The Affymetrix IDs is valid: 209047_at), AQP6/2L (The Affymetrix IDs is valid: 216219_at), *AQP4* (The Affymetrix IDs is valid: 226228_at), *AQP5* (The Affymetrix IDs is valid: 213611_at), *AQP8* (The Affymetrix IDs is valid: 206784_at), and *AQP10* (The Affymetrix IDs is valid: 1555338_s_at) mRNA were associated with poor OS in all gastric cancer patients HR = 1.55 (1.29–1.86), *P*=0.0000022, HR = 1.76 (1.47–2.09), *P*=1.70E-10, HR = 1.44 (1.16–1.79), *P*=0.0009, HR = 1.31 (1.07–1.61), *P*=0.0086, HR = 1.79 (1.51–2.13), *P*=1.90E-11 and HR = 1.45 (1.12–1.87), *P*=0.0043, respectively. On the other hand, overexpression of *AQP3* (The Affymetrix IDs is valid: 39248_at.), *AQP9* (The Affymetrix IDs is valid: 205568_at), and *AQP11* (The Affymetrix IDs is valid: 229526_at) mRNA were significantly associated with favorable OS in all gastric cancer patients, HR = 0.82 (0.69–0.97), *P*=0.023, HR = 0.67 (0.56–0.8), *P*=8.60E-06 and HR = 0.65 (0.52–0.82), *P*=0.00024, respectively. However, *AQP2* (The Affymetrix IDs is valid: 236630_at) mRNA expression did not show any correlation in the prognosis to all gastric cancer patients, HR = 1.24 (1–1.55), *P*=0.056 (Figure 3A–K).

### Prognostic correlation of AQP expression in gastric cancer patients with histological subtypes according to Laurens classification

Next, we evaluated the correlation between *AQPs* mRNA expressions according to distinct histological profiles based on Laurens classification. We determined the prognostic events for intestinal type (*n*=320) and diffuse type (*n*=241) gastric cancer patients (Table 1). The results revealed that *AQP0, AQP1, AQP2, AQP4, AQP6/2L, AQP8*, and *AQP10* mRNA expression in intestinal type gastric cancer patients were associated with unfavorable OS whereas, *AQP9* and *AQP11* mRNA expression were associated with favorable OS, nonetheless, *AQP3* and *AQP5* mRNA expression showed no correlation to prognosis in intestinal type gastric cancer. Consistently, high expression of *AQP2, AQP8, AQP9*, and *AQP11* mRNA were associated with longer OS in diffuse type gastric cancer, while high level of *AQP1* and *AQP10* mRNA expression were associated with poor OS. Notably, the remaining AQP submembers (*AQP0, AQP3, AQP4, AQP5*, and *AQP6/2L*) were not correlated with OS in diffuse type gastric cancer.

**Table 1:** Correlation of *AQPs* mRNA expression with histological subtypes according to Laurens classification of gastric cancer patients

AQP	Laurens classification	HR (95% CI)	Log-rank *P*	Cases
AQP0	Instestinal	2.11 (1.53–2.91)	0.0000032*	320
	Diffuse	0.86 (0.61–1.2)	0.37	241
AQP1	Instestinal	1.72 (1.18–2.5)	0.0044*	320
	Diffuse	1.73 (1.23–2.43)	0.0016*	241
AQP2	Instestinal	1.81 (1.25–2.62)	0.0015*	269
	Diffuse	0.7 (0.49–0.99)	0.045*	240
AQP3	Instestinal	0.86 (0.6–1.24)	0.42	320
	Diffuse	1.2 (0.85–1.71)	0.3	241
AQP4	Instestinal	1.7 (1.07–2.7)	0.024*	269
	Diffuse	1.34 (0.94–1.91)	0.099	240
AQP5	Instestinal	1.38 (0.98–1.94)	0.065	320
	Diffuse	1.18 (0.83–1.67)	0.36	241
AQP6/2L	Instestinal	2.1 (1.51–2.91)	0.0000058*	320
	Diffuse	0.8 (0.53–1.2)	0.28	241
AQP8	Instestinal	2.28 (1.66–3.13)	0.00000015*	320
	Diffuse	0.66 (0.46–0.95)	0.026*	241
AQP9	Instestinal	0.69 (0.48–0.99)	0.04*	320
	Diffuse	0.58 (0.41–0.83)	0.0024*	241
AQP10	Instestinal	1.78 (1.11–2.85)	0.016*	269
	Diffuse	1.54 (1.04–2.27)	0.03*	240
AQP11	Instestinal	0.57 (0.39–0.84)	0.0034*	269
	Diffuse	0.65 (0.46–0.92)	0.015*	240

*P<0.05.

### Prognostic correlation of AQPs expression in gastric cancer patients with gender difference

We further accessed expression of AQPs and its correlation to the prognosis of gastric cancer in accordance to gender difference (Table 2). We found that high expression of *AQP9* and *AQP11* mRNAs were associated with improved OS in male gastric cancer patients. However, high level of *AQP0, AQP1, AQP4, AQP5, AQP6/2L, AQP8*, and *AQP10* mRNA were significantly associated with decreased survival in male patients, while AQP2 and AQP3 showed no correlation. Similarly, high expression of *AQP3* and *AQP9* mRNA revealed improved OS in female patients, whereas AQP0, AQP1, AQP6/2L and AQP8 were associated with poor OS in gastric cancer. However, *AQP2, AQP4, AQP5, AQP10*, and *AQP11* mRNA overexpression in female patients showed no correlation to the prognosis of gastric cancer. Taken together, while comparing gender outcomes, only *AQP9* mRNA expression demonstrated longer OS in both male and female gastric cancer patients.

**Table 2:** Correlation of *AQPs* mRNA expression with gender difference of gastric cancer patients

AQPs	Gender	HR (95% CI)	Log-rank *P*	Cases
AQP0	Male	1.52 (1.21–1.91)	0.00024*	545
	Female	1.61 (1.12–2.31)	0.0094*	236
AQP1	Male	1.32 (1.06–1.63)	0.011*	545
	Female	1.82 (1.19–2.79)	0.005*	236
AQP2	Male	1.22 (0.9–1.63)	0.19	349
	Female	0.7 (0.45–1.11)	0.12	189
AQP3	Male	0.82 (0.66–1.01)	0.063	545
	Female	0.65 (0.46–0.92)	0.015*	236
AQP4	Male	1.43 (1.07–1.92)	0.016*	349
	Female	1.43 (0.91–2.23)	0.12	187
AQP5	Male	1.31 (1.01–1.7)	0.039*	545
	Female	1.24 (0.86–1.79)	0.24	236
AQP6/2L	Male	1.64 (1.33–2.03)	0.0000042*	545
	Female	2.36 (1.63–3.4)	0.0000023*	236
AQP8	Male	1.69 (1.36–2.1)	0.0000014*	545
	Female	2.25 (1.58–3.2)	0.0000036*	236
AQP9	Male	0.69(0.55–0.87)	0.0013*	545
	Female	0.55 (0.38–0.8)	0.0015*	236
AQP10	Male	1.66 (1.14–2.4)	0.0068*	349
	Female	1.32 (0.85–2.03)	0.21	187
AQP11	Male	0.54 (0.4–0.73)	0.00005*	349
	Female	0.69 (0.43–1.09)	0.11	187

*P<0.05.

### Prognostic correlation of AQPs expression in gastric cancer patients with different clinical stages

Furthermore, we obtained the data about each *AQP* mRNA expression and correlation of gastric cancer patients with all clinical stages (I, II, III, and IV) (Table 3). We noticed that high expression of *AQP0, AQP1, AQP8*, and *AQP10* mRNA were associated with worse prognosis in stages I and III. In addition, high *AQP2* mRNA expression was associated with poor OS in stage III. *AQP3* mRNA expression was associated with longer OS in stage I gastric cancer, however, it did not show any association with other stages. *AQP4* and *AQP6/2L* mRNA overexpression were correlated with poor OS in stage III gastric cancer. Similarly, high *AQP5* mRNA expression was associated with longer OS in stages I and IV gastric cancer patients. *AQP9* mRNA expression was also associated with favorable OS in stage I gastric cancer patients, as well as it showed improved OS in stage III gastric cancer patients. Eventually, *AQP11* mRNA high expression was significantly associated with stages III and IV gastric cancer patients.

**Table 3:** Correlation of *AQPs* mRNA expression with different clinical stages of gastric cancer patients

AQP	Clinical stages	HR (95% CI)	Log-rank *P*	Cases
AQP0	I	3.58 (1.33–9.64)	0.0072*	67
	II	0.66 (0.35–1.24)	0.19	140
	III	1.75 (1.32–2.34)	0.0001*	305
	IV	1.32 (0.87–1.98)	0.19	148
AQP1	I	8.44 (1.11–64.15)	0.014*	67
	II	1.46 (0.79–2.72)	0.23	140
	III	1.95 (1.4–2.7)	0.000047*	305
	IV	1.35 (0.87–2.1)	0.18	148
AQP2	I	0.55 (0.17–1.86)	0.33	62
	II	0.69 (0.33–1.46)	0.33	135
	III	1.46 (1.01–2.12)	0.044*	197
	IV	0.69 (0.45–1.06)	0.088	140
AQP3	I	0.35 (0.13–0.96)	0.034*	67
	II	0.57 (0.31–1.03)	0.06	140
	III	1.18 (0.89–1.58)	0.25	305
	IV	0.76 (0.51–1.14)	0.18	148
AQP4	I	2.3 (0.5–10.54)	0.27	62
	II	1.5 (0.8–2.8)	0.2	135
	III	1.88 (1.14–3.08)	0.011*	197
	IV	1.25 (0.84–1.87)	0.27	140
AQP5	I	0.36 (0.13–0.96)	0.033*	67
	II	1.79 (0.98–3.26)	0.056	140
	III	1.19 (0.88–1.6)	0.27	305
	IV	0.64 (0.42–0.96)	0.032*	148
AQP6/2L	I	1.86 (0.67–5.11)	0.22	67
	II	1.65 (0.9–3.04)	0.1	140
	III	1.94 (1.36–2.77)	0.00022*	305
	IV	1.18 (0.8–1.75)	0.41	148
AQP8	I	3.04 (1.04–8.85)	0.033*	67
	II	1.76 (0.95–3.27)	0.07	140
	III	1.7 (1.25–2.29)	0.00054*	305
	IV	1.43 (0.96–2.14)	0.078	148
AQP9	I	0.15 (0.03–0.67)	0.0043*	67
	II	1.26 (0.69–2.3)	0.44	140
	III	0.56 (0.42–0.74)	0.000053*	305
	IV	1.33 (0.88–2.02)	0.18	148
AQP10	I	7.03 (0.91–54.25)	0.03*	62
	II	0.82 (0.44–1.56)	0.55	135
	III	1.95 (1.18–3.24)	0.0083*	197
	IV	1.51 (0.97–2.33)	0.064	140
AQP11	I	2.66 (0.87–8.09)	0.075	62
	II	1.72 (0.91–3.25)	0.092	135
	III	0.59 (0.4–0.88)	0.0081*	197
	IV	0.53 (0.35–0.79)	0.0018*	140

*P<0.05.

### Prognostic correlation of AQPs expression with *HER2* gene

Table 4 revealed that high level of *AQP0, AQP4, AQP6/2L*, and *AQP8* mRNA expression with both HER2 positive and negative genes were associated with poor OS in gastric cancer patients. In addition, *AQP1* and *AQP2* mRNA expression in gastric cancer patients with HER2 negative gene were associated with unfavorable OS. Whereas, *AQP10* and *AQP5* mRNA overexpression was associated with poor prognosis with HER2 positive. Additionally, *AQP9* and *AQP11* mRNA expression with negative HER2 was associated with longer survival rate in gastric cancer patients. Likewise, higher *AQP3* mRNA expression was associated with favorable OS in gastric patients both in HER2 positive and negative patients.

**Table 4:** Correlation of *AQPs* mRNA expression with HER2 status of gastric cancer patients

AQP	HER2 status	HR (95% CI)	Log-rank *P*	Cases
AQP0	Negative	1.5 (1.18–1.92)	0.001*	532
	Positive	1.47 (1.13–1.9)	0.0037*	344
AQP1	Negative	1.36 (1.08–1.7)	0.0076*	532
	Positive	1.21 (0.92–1.59)	0.18	344
AQP2	Negative	1.34 (1.02–1.77)	0.035*	429
	Positive	0.76 (0.49–1.19)	0.23	202
AQP3	Negative	0.79 (0.63–0.99)	0.037*	532
	Positive	0.73 (0.54–0.98)	0.033*	344
AQP4	Negative	1.57 (1.2–2.06)	0.00097*	429
	Positive	1.52 (1.01–2.28)	0.044*	202
AQP5	Negative	1.22 (0.94–1.58)	0.14	532
	Positive	1.33 (1.02–1.73)	0.035*	344
AQP6/2L	Negative	2.1 (1.67–2.65)	0.00000000009*	532
	Positive	1.38 (1.06–1.79)	0.016*	344
AQP8	Negative	1.71 (1.37–2.15)	0.0000019*	532
	Positive	1.86 (1.36–2.55)	0.000073*	344
AQP9	Negative	0.53 (0.42–0.67)	0.000000057*	532
	Positive	1.17 (0.9–1.52)	0.23	344
AQP10	Negative	1.24 (0.9–1.7)	0.18	429
	Positive	2.04 (1.25–3.35)	0.0039*	202
AQP11	Negative	0.59 (0.45–0.78)	0.00013*	429
	Positive	0.74 (0.49–1.12)	0.16	202

*P<0.05.

### Prognostic correlation of AQPs expression with different treatment strategy

The database results of individual AQPs (Table 5) correlation with different treatment strategy revealed that high expression of AQP0 and AQP6/2L were associated with poor OS in gastric cancer patients who received only surgery. Consequently, *AQP1* mRNA expression was associated with better OS in gastric patients treated with 5 FU-based adjuvant chemotherapy, whereas it was related with significantly poor prognosis with other adjuvant therapy. High *AQP5* and *AQP10* mRNA levels were also associated with better OS with 5 FU treatment, however, it did not illustrate any relation with other therapeutic strategies. *AQP8* mRNA expression on the other hand was associated with favorable OS with other adjuvant chemotherapy, but significantly correlated with poor OS to surgery only treatment. In addition, AQP9 high mRNA level was associated with favorable OS to surgery alone, whereas with 5 FU-based adjuvant chemotherapy showed remarkably poor OS, and no correlation was observed with other adjuvants. *AQP11* mRNA expression was associated with better OS in gastric cancer patients with surgery alone and other adjuvant chemotherapeutic treatment. However, *AQP2, AQP3*, and *AQP4* high mRNA level were not associated with the prognosis of gastric cancer patients to any of the treatment methods (surgery alone, 5 FU adjuvant, and other adjuvant).

**Table 5:** Correlation of *AQPs* mRNA expression with various treatment strategies of gastric cancer patients

AQP	Treatment	HR (95% CI)	Log-rank *P*	Cases
AQP0	Surgery alone	1.4 (1.03–1.9)	0.032*	380
	5 FU-based adjuvant	0.79 (0.56–1.12)	0.18	153
	Other adjuvant	0.56 (0.19–1.66)	0.29	76
AQP1	Surgery alone	1.34 (0.99–1.81)	0.06	380
	5 FU-based adjuvant	0.58 (0.4–0.85)	0.0052*	153
	Other adjuvant	3.31 (1.37–8)	0.0048*	76
AQP2	Surgery alone	0.81 (0.59–1.11)	0.18	380
	5 FU-based adjuvant	1.89 (0.55–6.51)	0.3	34
	Other adjuvant	0.57 (0.24–1.37)	0.2	76
AQP3	Surgery alone	0.87 (0.65–1.16)	0.33	380
	5 FU-based adjuvant	1.24 (0.84–1.82)	0.28	153
	Other adjuvant	1.91 (0.64–5.71)	0.24	76
AQP4	Surgery alone	1.23 (0.89–1.69)	0.2	380
	5 FU-based adjuvant	0.41 (0.12–1.4)	0.14	34
	Other adjuvant	1.88 (0.77–4.62)	0.16	76
AQP5	Surgery alone	1.34 (0.98–1.84)	0.067	380
	5 FU based adjuvant	0.69 (0.48–1)	0.048*	153
	Other adjuvant	0.48 (0.16–1.44)	0.18	76
AQP6/2L	Surgery alone	1.38 (1.02–1.88)	0.036*	380
	5 FU-based adjuvant	1.47 (0.98–2.21)	0.059	153
	Other adjuvant	0.42 (0.12–1.43)	0.15	76
AQP8	Surgery alone	1.49 (1.11–2)	0.0081*	380
	5 FU-based adjuvant	1.29 (0.9–1.84)	0.16	153
	Other adjuvant	0.38 (0.16–0.9)	0.023*	76
AQP9	Surgery alone	0.72 (0.53–0.96)	0.026*	380
	5 FU-based adjuvant	1.56 (1.1–2.22)	0.011*	153
	Other adjuvant	2.04 (0.84–4.92)	0.11	76
AQP10	Surgery alone	1.41 (0.99–2.02)	0.057	380
	5 FU-based adjuvant	0.26 (0.08–0.82)	0.015*	34
	Other adjuvant	1.84 (0.61–5.5)	0.27	76
AQP11	Surgery alone	0.72 (0.53–0.98)	0.035*	380
	5 FU-based adjuvant	3.89 (0.89–16.95)	0.052	34
	Other adjuvant	0.26 (0.09–0.72)	0.0053*	76

*P<0.05.

## Discussion

Over the past decades, multiple studies have presented the significant role of ion channels and water carriers in gastric cancer [24,25]. So far, screening the expression profile of AQPs transmembrane proteins have revealed 13 different subtypes expression between cancer and adjacent normal tissues. The principle of the current study was to identify the research community with a well-annotated resource of proteins expressed in gastric cancer with higher quality. This could improve our understanding of general gastric biology and guide to find novel gastric cancer biomarker. To achieve this goal, we mapped our outcomes to two publicly available resources: HPA (www.proteinatlas.org) and Kaplan–Meier survival plot (http://kmplot.com/analysis/). Using HPA database, we noticed aberrant protein expression of individual AQP genes both in normal and gastric cancer tissues have crucial role to predict cancer progression. Likewise, KM plot database showed that ten AQPs subtypes were associated with the prognosis of all gastric cancer patients. In which, we revealed *AQP3, AQP9*, and *AQP11* mRNA expression were associated with improved OS in all gastric cancer patients especially with intestinal subtypes, whereas *AQP0, AQP1, AQP4, AQP5, AQP6/2L AQP8*, and *AQP10* mRNA expression were associated with poor OS in all gastric cancer patients. Moreover, similar significant predictive role of *AQPs* mRNA were detected in the prognosis of distinct clinocapthological study of gastric cancer patients; such as Laurens classification, clinical stage, HER2 status, and different treatment strategy.

*AQP0* mRNA expression have been identified in retina, liver, and Sertoli cells of testis and abundantly expressed in the lens fiber cells [26]. Moreover, Shen et al. [15] found low *AQP0* mRNA expression in human gastric carcinoma and corresponding normal tissue through RT-PCR method. Studies about the correlation of *AQP0* mRNA with gastric cancer have not been published yet, however, in the present analysis using the database study, we revealed that *AQP0* mRNA was associated with poor survival rate in all cancer patients, especially with intestinal type both in male and female, and as well as in clinical stages I and III. Whereas, in HPA database study, we did not find any results regarding AQP0 protein expression in gastric cancer.

Increased AQP1 in tumor cells has shown to enhance metastatic potential and raise local infiltration [10,11]. In gastric cancer, high level of AQP1 in tumor cells and tumor vessels was associated with the development and promotion of gastric tumors and lymphatic metastasis [27]. Sun et al. [28] demonstrated that AQP1 expression is associated with poor prognosis in gastric adenocarcinoma and, thus can be a predictive prognostic marker. Consistently, our results using HPA database showed that AQP1 protein was highly expressed in gastric cancer tissues at cytoplasm, whereas it was not detected in normal gastric tissues. This comparison of tumor and adjacent non-tumor tissues suggested that the dysregulation of AQP1 was associated with tumorigenesis in gastric cancer. Subsequently, high *AQP1* mRNA expression both in male and female was also correlated to poor OS in all the gastric cancer patients including intestinal and diffuse types. Moreover, we observed that increased *AQP1* mRNA expression was significantly associated with higher mortality rate in stages I and III gastric cancer patients.

AQP2 is mostly identified in the cells of kidney collecting ducts responsible for the passage of water molecule [29]. Distinct role of AQP2 expression in cancer patients including gastric cancer remains largely unknown. Through HPA database, we observed that AQP2 protein was not expressed, both in normal and gastric cancer tissues. Additionally, from mRNA analysis, *AQP2* mRNA expression showed null relation to the prognosis in all gastric cancer patients, clinical stage (stages I, II and IV) of both male and female population. Notably, prognostic evaluation according to histopathology reports revealed that gastric cancer patients with intestinal subtype at stage III with positive *AQP2* mRNA were associated with poor survival rate.

AQP3 is frequently studied aquaporin subtype in gastric cancer patients. Previous study has shown that AQP3 expression was highly associated with gastric cancer compared with normal cells, aggravating carcinogenesis and progression with lymph node metastasis and lymphovascular invasion [15]. Chen et al. [14] documented that expression of AQP3 could promote epithelial–mesenchymal transition (EMT) in human gastric cancer through p13K/AKT/Snail signaling pathway leading to poor prognosis. In this report, HPA database outcomes suggested markedly increased AQP3 protein expression in normal gastric tissue, and as well as in gastric cancer tissue. Consequently, further analysis via KM plot exhibited that increased expression of *AQP3* mRNA had no correlation with histological subtypes, male patients, and advanced clinical stages. However, overexpression was associated with improved survival rate in all gastric cancer patient mostly in females and early stage (stage I) tumors. Although AQP3 has significant role in gastric cancer, presented conflicting outcomes may be related with different population variation, different media to determine cut-off points, and different follow-up periods, thus AQP3 expression may need further investigation with larger trials and longer follow-up with standard methodology.

The expression of AQP4 has been suggested as a marker of normal proliferating gastric epithelial cells [30]. Shen et al. [15] using IHC study illustrated that AQP4 expression was present in the membrane of chief cells and parietal cells of normal gastric mucosa. Interestingly, IHC analysis through HPA database also demonstrated significant AQP4 protein expression in normal tissues compared with adjacent gastric cancer tissue. Consistently, the prognostic correlation of *AQP4* mRNA expression in gastric cancer showed significant poor prognosis in all gastric cancer patients primarily in stage III, as well as in male intestinal gastric cancer patients.

Furthermore, AQP5 up-regulation has additionally been noticed in human gastric cancer [31]. Up-regulation of AQP5 has been suggested to play an important role in the differentiation, tumorigenesis, and progression of gastric cancer [32]. Subsequently, a similar effect was detected for AQP5 protein expression in gastric cancer patients through HPA database. In particular, IHC staining was decreased in normal tissues, while medium staining was observed in gastric cancer tissues. On the other hand, the survival analysis demonstrated that *AQP5* mRNA expression was associated with poor OS to all gastric cancer patient especially in male patients. Therefore, these outcomes imply that accumulation of elevated AQP5 protein and mRNA may result in tumorigenesis and tumor progression.

AQP6 is also known as AQP6/2L, which exhibits low water permeability and exclusively detected in acid secreting intercalated cells of kidney collecting ducts regulating renal acid base [33]. Only few studies have investigated AQP6 expression in tumor cells. In a recent preclinical study, AQP6 expression has been identified in rat gastrointestinal epithelium [34], however, the prognostic relation of AQP6 in human gastric cancer have rarely been reported in previous studies. From the present database study, we explored that overexpression of *AQP6* mRNA was associated with increased risk of mortality in all male and female gastric patients mainly with the intestinal type and clinical stage III. Notably, we did not find any AQP6 protein expression both in normal and gastric cancer tissues in HPA database.

*AQP8* mRNA expression in gastrointestinal tract mostly expressed in normal colonic tissue compared with adjacent adenomas, carcinomas, and cancer cell lines [35]. Previous study has suggested that *AQP8* mRNA was significantly lower in HCC compared with corresponding normal cells, confirming that AQP8 is a promising target for HCC therapy and useful biomarkers [36]. But its role in prognosis in gastric cancer is barely explored. According to our database results medium expression trend was exhibited in gastric cancer tissues compared with null expression in normal tissues. In addition, elevated expression of *AQP8* mRNA in all gastric cancer patients was associated with poor OS, prominently in both male and female intestinal type gastric cancer patients, and similarly in stages I and III patients. But, it was associated with better OS to diffuse type gastric cancer patients.

Huang et al. [37] showed that high expression of AQP9 was correlated with improved disease-free survival (DFS) and increased chemosensitivity in stage III colorectal cancer. Nonetheless, *AQP9* mRNA expression and its role in gastric cancer remains to be clarified along with tumor marker and suppression characteristics. In the present study, we did not find any significant protein expression from HPA investigations both in normal and gastric cancer tissues. Additionally, *AQP9* mRNA analysis showed that overexpression was linked with favorable OS in all gastric cancer patients including both intestinal and diffused type. Furthermore, both male and female gastric cancer patients presented with high *AQP9* mRNA were also associated with longer survival rate especially in stages I and III. Therefore, positive *AQP9* mRNA expression in gastric cancer can be a good prognostic marker.

AQP10 is an aquaglyceroporin that transmits water, glycerol, and urea, which is abundantly expressed in the small intestine and colon [38]. Evidently, the association between AQP10 in gastric cancer is not known yet. Indeed, in the HPA database, differentials in AQP10 protein expression between normal and gastric cancer tissue were also not detected. However, from the ‘KM plotter’ database, we determined that *AQP10* mRNA expression was associated with poor survival in all gastric cancer patients involving both histological subtypes (intestinal and diffuse), male gender and in stages I and III patients.

Previously published study has suggested exclusive expression of *AQP11* mRNA primarily in healthy human duodenum tissues and proximal tubules of kidney [38]. The crucial function of this aquaporin in relation to cancer still remains to be elucidated. In this report, observation from HPA database revealed that AQP protein high expression was documented in normal tissues compared with medium expression in adjacent gastric cancer tissues. Thus, it illustrates that future rigorous investigations in protein expression with larger samples may differentiate its potentiality as cancer biomarker. However, the survival analysis demonstrated that *AQP11* mRNA expression was significantly associated with improved OS in all gastric patients involving both intestinal and diffuse histological subtypes especially in advanced stage male gastric cancer patients.

HER 2 is a tyrosine receptor kinases (RTKs) belonging to the family of EGFR that is located on chromosome 17q21, and plays an essential role in cell survival and proliferation [39]. High expression of HER2 receptor as a prognostic and predictive biomarker is becoming noticeable even in gastric cancer, although few controversies are present regarding its association with clinical features of tumor. Approximately 10–30% gastric cancers showed positive HER2 expression [40]. Wang et al. [41] in a meta-analysis reported that HER2 positive expression was associated with male gender, intestinal type, and well/moderate cell differentiation. In the present study, we documented that high mRNA level of AQP0, AQP4, AQP2L/6, and AQP8 were associated with worse OS in either HER2 positive or HER2 negative gastric cancer patients. In addition, *AQP10* and *AQP5* mRNA expression with positive HER2 and AQP1 and AQP2 mRNA with negative HER2 were associated with poor survival in gastric cancer patients. Intriguingly, *AQP3* mRNA expression was associated with better OS with positive and negative HER2 in gastric cancer patients, whereas *AQP9* and *AQP10* mRNA levels were associated with better OS with negative HER2.

A number of recent studies have revealed that great roles of human AQPs in tumor proliferation, angiogenesis, and metastasis, and its cross-talk with various signaling pathways have developed a linkage for drug target of gastric cancer. AQPs target inhibitors consisting cysteine-reactive heavy metal-based inhibitors, small molecule inhibitors which inhibit AQPs expression or AQP-mediated water permeation, and monoclonal AQP specific antibody have been established and proven [42]. Apart from the detoxification pathways, AQPs are seen to confer chemosensitivity and resistance in some tumors by allowing the transport of metalloids into cells [43,44]. AQPs are closely associated with other transmembrane transport channels and showed an essential function in chemosensitivity, drug metabolism, and cell apoptosis through water permeability regulations [45]. Therefore, AQP’s expression in gastric cancer could also impact the therapeutic efficiency and prognosis. In the present study, we observed that *AQP1, AQP5*, and *AQP10* mRNA expression were correlated with improved survival rate with protective effect in those who received 5 FU-based adjuvant chemotherapy in gastric cancer patients and *AQP9* mRNA higher level was correlated with better OS in patients who underwent only surgery. Furthermore, *AQP8* and *AQP11* mRNA expression in other adjuvant chemotherapy showed better OS in gastric cancer patients. Nonetheless, AQP0, AQP6, and *AQP8* mRNA expression with surgery alone and *AQP9* mRNA with 5 FU chemotherapy were associated with significantly increased risk of low survival rate in gastric cancer patients.

## Conclusion

The present study shows that differential expression of AQPs protein and mRNA are significantly associated in predicting the prognosis of gastric cancer patients. Comprehensive exploration of the individual AQPs from the database suggested that mRNA expression of AQP3, AQP9, and AQP11 were correlated with improved OS in all gastric patients, whereas *AQP0, AQP1, AQP4, AQP5, AQP6, AQP8*, and *AQP10* mRNA higher expression were associated with poor OS. Moreover, AQPs showed critical prognostic events in gastric cancer patients with different clinicopathological features such as Laurens classification, gender, pathological grade, clinical stage, HER2 status, and different choices of treatments. So far, AQPs are unique membrane proteins strongly associated with cancer development and progression. Targetted pharmacological modulation of water and solute transport using AQPs may implement its potential therapeutic interventions in cancer patients. Therefore, a deeper understanding of molecular mechanisms may lead us to the future discovery of AQPs as an effective targetted prognostic marker and a novel treatment agent in gastric cancer patients.

## Abbreviations

AQP: aquaporin
CAB: commercially available antibody
EGFR: epidermal growth factor receptor
HCC: hepatocellular carcinoma
HER2: human epidermal growth factor receptor 2
HPA: human protein atlas
HR: hazard ratio
IHC: immunohistochemistry
KM plotter: Kaplan–Meier plotter
RT-PCR: reverse transcription polymerase chain reaction
TNM: tumor lymph node metastasis
OS: overall survival
5 FU: 5-fluorouracil
